# The Metreleptin Effectiveness and Safety Registry (MEASuRE): concept, design and challenges

**DOI:** 10.1186/s13023-023-02714-5

**Published:** 2023-05-26

**Authors:** Morey W Haymond, David Araújo-Vilar, John Balser, James H Lewis, Ruth Louzado, Carla Musso, Julia von Schnurbein, Martin Wabitsch, on behalf of the MEASuRE group

**Affiliations:** 1 Clinical Care Center, Texas Children’s Hospital, Baylor College of Medicine, 6701 Fannin St., 11th floor, Houston, TX 77030 USA; 2 Thyroid and Metabolic Diseases Unit, Centro de Investigación en Medicina Molecular y Enfermedades Crónicas (CIMUS)-IDIS, School of Medicine, Universidade de Santiago de Compostela, Avda. Barcelona 3, Santiago de Compostela, 15707 Spain; 3 Veristat LLC, 134 Turnpike Rd #200, Southborough, MA 01772 USA; 4 MedStar Georgetown University Hospital, Washington, DC USA; 5 Amryt Pharmaceuticals DAC, 45 Mespil Road, Dublin 4, Ireland; 6 Diabetes section, Fundacion Favaloro, Buenos Aires, Argentina; 7 Division of Paediatric Endocrinology and Diabetes, Department of Paediatrics and Adolescent Medicine, Centre for Rare Endocrine Disorders, Ulm University Medical Centre, Eythstraße 24, 89075 Ulm, Germany

**Keywords:** Lipodystrophy, Leptin, Metreleptin, Registry, Real-world data

## Abstract

**Background:**

Metreleptin, a recombinant analog of human leptin, is an approved therapy, adjunct to diet, to treat the metabolic complications of leptin deficiency in patients with lipodystrophy – a group of rare diseases characterized by a paucity of adipose tissue. MEASuRE (Metreleptin Effectiveness And Safety Registry) is a post-authorization, voluntary registry that gathers long-term safety and effectiveness data on metreleptin. Here, we present the aims and evolution of MEASuRE.

**Methods:**

MEASuRE was established to collect data from patients receiving commercially supplied metreleptin in the United States (US) and European Union (EU). MEASuRE aims to determine the incidence and severity of safety events and describe the clinical characteristics and therapeutic outcomes in the metreleptin-treated population. A key feature of MEASuRE is that it accumulates data from different sources to meet post-authorization objectives. US data are received directly from treating physicians via a contract research organization-mediated electronic data capture system. In the EU, data are received via the European Registry of Lipodystrophies managed by the European Consortium of Lipodystrophies (ECLip), a platform established by researchers and physicians to advance the knowledge of lipodystrophy. MEASuRE complies with applicable regulatory requirements governing privacy, and the storage, management, and access of data.

**Results:**

Leveraging processes, infrastructure, and data from the ECLip registry presented several challenges that were addressed during MEASuRE’s development, including the expansion of the ECLip registry to accommodate MEASuRE-specific data elements, extensive data matching processes to ensure data consistency regardless of source, and rigorous data validation following the amalgamation of global data. Through the support of ECLip, MEASuRE is now a fully operational registry with the capacity for gathering and integrating standardized US- and EU-derived data. As of 31st October 2022, 15 US and four EU sites have participated in the MEASuRE, enrolling 85 patients globally.

**Conclusions:**

Our experiences show that a post-authorization product registry can be successfully integrated into an existing patient registry. We propose that, through collaboration with existing registries and use of their established resources, patient enrolment timelines and data collection for new registries can be expedited. The learnings presented here may be applicable to other registries with similar objectives.

**Trial registration:**

NCT02325674; Registered 25 December 2014 - Retrospectively registered’. https://clinicaltrials.gov/ct2/show/NCT02325674.

## Introduction

Lipodystrophy syndromes are a rare, life-limiting, heterogeneous group of diseases characterized by a complete or partial deficiency of adipose tissue and are associated with a series of metabolic complications and organ system abnormalities [1]. Lipodystrophy syndromes are typically classified according to the underlying disease etiology (i.e., inherited or acquired) and the extent of adipose tissue loss (generalized or partial), giving rise to four main categories: congenital generalized lipodystrophy (CGL), acquired generalized lipodystrophy (AGL), familial partial lipodystrophy (FPLD), and acquired partial lipodystrophy (APL) [2]. To date, up to 50 clinically heterogeneous lipodystrophy subtypes have been identified [3], illustrating the complex pathophysiology of these diseases. CGL is classically caused by variants in the *AGPAT2*, *BSCL2*, *CAV1*, *CAVIN/PTRF* and *PPARG* genes; however, other genes are reported to be associated with generalized adipose tissue loss [3, 4]. Although variants in the *LMNA* gene account for most FPLD cases with a known genetic etiology, several other genes have been associated with FPLD including cases presenting with symmetric lipomatosis, progeroid features, and more complex syndromes. For some patients with FPLD, the genetic etiology has not yet been identified [3, 5].

Except for cases due to human immunodeficiency virus (HIV), lipodystrophy syndromes are extremely rare [2]. In 2011, a review of published cases estimated the prevalence of genetic lipodystrophies at less than one in a million with a further 250 APL and 100 AGL cases reported in the literature [6]. More recently, structured interrogation of international electronic medical record (EMR) databases reported the global prevalence of lipodystrophy at 1.3–4.7 cases per million, with estimates of 0.23 cases per million and 2.84 cases per million for generalized and partial forms, respectively. In the same analysis, literature searches involving the European population reported a prevalence of 0.96 cases per million for generalized lipodystrophy (GL) and 1.67 cases per million for partial lipodystrophy (PL) [7]. It is acknowledged that lipodystrophy is underdiagnosed, especially partial forms of the disease, and that the true clinical prevalence may be under-represented in the literature [1, 3, 7, 8]. For example, an analysis of US EMRs performed by Gonzaga-Jauregui et al. (2020) reported the genetic prevalence of lipodystrophy as 1 in 7,000 [8].

The deficiency of adipose tissue associated with lipodystrophy can result in ectopic lipid accumulation in organs such as the liver and muscles, often leading to severe insulin resistance, hypertriglyceridemia, and non-alcoholic fatty liver disease (NAFLD). Consequently, many patients with GL and PL present with a range of metabolic and organ abnormalities including diabetes, cardiovascular disease, hepatic steatosis, nephropathy, and pancreatitis. Furthermore, patients with lipodystrophy, especially GL, are typically hyperphagic due to the reduced levels of the adipokine, leptin, which is a key physiological regulator of satiety, energy homeostasis, insulin action and lipid metabolism [1, 2, 6, 9]. Lipodystrophy may also result in other physiological conditions such as reproductive dysfunction and impaired immune responses as well as less well-documented complications including psychological problems, fatigue, pain, and reduced quality of life [1, 2, 6, 9–11].

Treatment of lipodystrophy typically focuses on the improvement or prevention of metabolic dysfunction and aims to reduce the long-term risks associated with the complications of diabetes, high triglyceride levels, severe insulin resistance and end-organ damage. As ectopic lipid accumulation and insulin resistance are driven by the impaired ability to store excess calories in adipocytes, the management of lipodystrophy also includes the reduction of caloric intake. This approach, however, is often challenging in hyperphagic patients with low leptin levels [1, 2, 5, 12].

Recombinant human methionyl leptin (metreleptin), administered via subcutaneous injection, is the only medicinal product currently approved for the treatment of the metabolic complications associated with lipodystrophy [13]. In the United States (US), metreleptin was approved by the Food and Drug Administration (FDA) in February 2014, as an adjunct to diet as replacement therapy to treat the complications of leptin deficiency in patients with CGL and AGL [14]. In the European Union (EU), metreleptin was approved in July 2018 by the European Medicines Agency (EMA) as an adjunct to diet as replacement therapy to treat the complications of leptin deficiency in adults and children aged ≥ 2 years with confirmed CGL or AGL or in adults and children aged ≥ 12 years with confirmed FPLD or APL for whom standard treatments have not achieved adequate metabolic control [15]. These regulatory approvals were predominantly based on combined data from an open-label pilot study conducted at the US National Institutes of Health (NIH) (NIH 991265 [ClinicalTrials.gov identifier: NCT00005905]) and its long-term extension (NIH 20010769 [NCT00025883]), which evaluated the long-term efficacy and safety of metreleptin in patients with GL and PL [16, 17]. However, it is recognized that the low prevalence of lipodystrophy limits published studies involving metreleptin to small sample sizes. Furthermore, the interpretation of the therapeutic effects of metreleptin may be further challenged by the considerable phenotypic heterogeneity of lipodystrophy [9].

One approach to address the paucity of information regarding rare diseases, including lipodystrophy, is to use patient registries—organized systems designed to collect and combine standardized longitudinal clinical data to identify specified outcomes for a population defined by a particular disease, condition, or exposure [18–20]. Patient registries are generally categorized according to how their populations are defined; for example, disease registries are defined by patients having a particular disease regardless of exposure to any medicinal product, while product registries collect data from particular patient populations receiving specific medicinal products [18, 21, 22].

The potential benefits of registries are manifold; they can improve the understanding of the natural history, diagnosis, and pathogenesis of rare diseases, as well as providing valuable data from real-world settings where the clinical effects and safety of therapeutic agents can be assessed. They can also identify patient cohorts that may be suitable for enrolment into clinical studies, and through pooling, provide sufficient data to power statistical analyses [22–24]. Indeed, the broad contribution of patient registries to rare diseases is advocated by recent initiatives published by the FDA and EMA [18, 25–27]. In support of this, the approval of metreleptin by the FDA and EMA was contingent on the establishment of a post-authorization registry to collect and analyze longitudinal safety and effectiveness data from patients treated with metreleptin in routine clinical practice. This approach allows data obtained from clinical studies to be supplemented with real-world evidence and has been used for the approval of many therapies for rare diseases and other disease types [22]. Consequently, the Metreleptin Effectiveness And Safety Registry (MEASuRE) was established following FDA approval of metreleptin to gather data from US patients. The registry was later expanded to EU patients following the approval of metreleptin by the EMA.

In designing the approach to the EU post-authorization requirement, the then sponsor of MEASuRE, Aegerion Pharmaceuticals (acquired by Amryt Pharmaceuticals in 2019), opted against engaging each participating European site on an individual basis for data collection via a central electronic data capture system, as per the approach adopted in the US. Instead, the sponsor sought collaboration with the European Consortium of Lipodystrophies (ECLip) to leverage data from eligible patients existing within their European Registry of Lipodystrophies (the ECLip registry). The ECLip registry was established in 2016 to collect and combine clinical and molecular data related to lipodystrophy syndromes from academic and clinical centers across Europe and neighboring countries [28]. This registry adheres to current EU recommendations, such as FAIR principles, ensuring that data and metadata are ‘findable, accessible, interoperable and reusable’ [29] and is affiliated to the European Registries for Rare Endocrine Conditions (EuRRECa) [30]. In the case of MEASuRE, it was envisaged that the collaboration with ECLip would avoid duplication of workload at the level of participating sites and would improve the efficiency of patient enrolment and data collection.

Here, we describe the aims, design, governance and functioning of MEASuRE. We also describe the history and evolution of MEASuRE and detail the harmonization of the US and EU objectives. Importantly, we detail the collaboration between the sponsor of MEASuRE and ECLip, which was pivotal in facilitating data collection from EU patients. Finally, we share our experiences on the challenges encountered and surmounted during the registry’s development, such that these learnings can be used to support the establishment of other patient registries, particularly those involving the management and treatment of rare diseases.

## Methods

MEASuRE (ClinicalTrials.gov: NCT02325674) is a non-interventional, multicenter, prospective, observational, voluntary registry of patients treated with commercial metreleptin therapy as part of standard clinical practice in the US and EU. The registry was established as a post-authorization requirement and specific obligation following the approval of metreleptin by the FDA and EMA, respectively.

### Ethics statement

MEASuRE is conducted in accordance with International Society for Pharmacoepidemiology Guidelines for Good Pharmacoepidemiology Practices [31] and with the standards of Good Clinical Practice as defined by the International Council for Harmonisation of Technical Requirements for Pharmaceuticals for Human Use [32]. The registry also complies with applicable federal and local regulations, and with the ethical principles originating from the Declaration of Helsinki [33]. In the US, all necessary institutional review board approvals were obtained, and in the EU, all necessary ethics committee approvals at a country and site level were obtained.

### Aim and objectives

MEASuRE aims to collect information regarding the long-term use of metreleptin. Its primary objective is to supplement data from clinical trials by determining the real-world incidence and severity of adverse events (AEs) in patients prescribed metreleptin. As a secondary objective, MEASuRE collects available data to describe the demographic and clinical characteristics of metreleptin-treated patients and to monitor longitudinal changes in routine laboratory measurements that could be inferred as effectiveness endpoints. MEASuRE also provides the opportunity to obtain further data on the incidence rates of specific safety events and other clinical parameters involving metreleptin-treated patients where current information is sparse. The full list of primary, secondary, and exploratory endpoints of MEASuRE is presented in Table 1.

**Table 1:** Primary endpoints, secondary endpoints and exploratory endpoints collected for MEASuRE

Primary objectives	To determine the incidence and severity of the following safety events in patients prescribed metreleptin in normal clinical practice: • Acute pancreatitis associated with the discontinuation of metreleptin; and all cases of fatal or necrotizing pancreatitis • Hepatic adverse events • Hypoglycemia stratified by severity and concomitant antidiabetics dose modifications • Hypersensitivity reactions • Serious and severe infections, including serious infections resulting in hospitalization and death • Loss of efficacy, potentially due to ADAs with blocking activity • New diagnoses of autoimmune disorders (for instance, autoimmune hepatitis, glomerulonephritis, lupus erythematosus, antiphospholipid antibody syndrome, rheumatoid arthritis) • Exacerbation of existing autoimmune disorders • All cancers (excluding non-melanoma skin cancer) by cancer type • Exposed pregnancies and pregnancy outcomes stratified by planned or unplanned • All-cause deaths (including causes of death) • Medication errors
Secondary objectives	• To describe the overall demographic and clinical characteristics, and metreleptin exposure in all patients treated with metreleptin (pattern of metreleptin use) • To describe routine laboratory measurements that could be inferred as effectiveness endpoints (including HbA1c, FPG, and TG) over time
Exploratory objectives	• Use in pregnancy and lactation • Use in elderly • Effect of metreleptin on brain development • Effect of metreleptin on bone metabolism • Effect of metreleptin on sexual maturation (Tanner staging) • Neuroendocrine parameters and levels of the following hormones: testosterone, estradiol, LH, FSH and free T3 and T4 • Contingent on study sample size, the study will also estimate the incidence rate of the primary outcomes of interest by patient characteristics • In patients with results from immunogenicity testing, the incidence of ADAs with blocking activity will be estimated

ADA, antidrug antibodies; FPG, fasting plasma glucose; FSH, follicle stimulating hormone; HbA1c, glycated hemoglobin; LH, luteinizing hormone; T3, triiodothyronine; T4, thyroxine; TG, triglycerides

### Governance of MEASuRE

MEASuRE is governed by its sponsor, Amryt Pharmaceuticals. The sponsor is responsible for all aspects of data collection, management and retention, data quality control, and data analysis as well as the training of staff from participating sites, as appropriate. The sponsor also provides the resources for study implementation and conducting analyses (including the preparation of scientific reports) involving data from the registry in a manner that meets regulatory and methodological standards.

A steering committee for MEASuRE has been established and includes clinicians and scientists with expertise in the areas of lipodystrophy and metabolic disease, epidemiology, endocrinology, hepatology, and biostatistics. Through collaboration with ECLip, data from EU patients are collected into MEASuRE via the ECLip registry (detailed below); thus, the MEASuRE steering committee was broadened to include ECLip registry board members not from participating EU sites, to ensure balanced communication and global representation. Steering committee members provide subject matter expertise for the MEASuRE program and are responsible for reviewing the data from the registry over time. Based on their review, the steering committee make continuous recommendations to the sponsor regarding the registry. They also assist in study design, study execution and interpretation, and in publication planning and manuscript review.

### Data ownership

In the US, data ownership for MEASuRE lies with the registry sponsor. For data originating from the ECLip registry, ownership resides with the patients and their treating physicians as appropriate for a patient registry [30].

### Study population

Patients eligible for inclusion in MEASuRE are those treated with at least one dose of commercially supplied metreleptin at sites in the US and in EU countries where metreleptin is reimbursed, and where their prescribing physician is a MEASuRE investigator. Eligible patients do not need to be taking metreleptin at the time of enrolment; they can be treated with at least one dose of commercial metreleptin at any time before registry participation. All treatment decisions, including visit frequency, are made at the discretion of the patient’s treating physician, and are not mandated by the MEASuRE protocol.

The inclusion criteria for MEASuRE are broad with limited exclusion criteria to best represent the population of patients taking metreleptin as per usual clinical practice. Inclusion criteria for registry enrolment are patients diagnosed with GL (US), or patients diagnosed with GL or PL (EU) who are, or who have been, treated with commercial metreleptin as part of their clinical care, and who have provided written consent before enrolment into the registry, and who have finished their participation in metreleptin clinical studies and are continuing or restarting treatment with metreleptin through commercial supply.

Patients receiving metreleptin off-label may be included in the registry, except for patients from Germany who must be treated in accordance with the EMA-approved indication for metreleptin [15]. Patients with localized and HIV-associated lipodystrophy and patients currently treated with an investigational agent as part of a clinical trial are excluded from the registry.

### Prescribing physician enrolment

Physicians who prescribe metreleptin are encouraged to invite their eligible patients to participate in MEASuRE. This enables the collection and assessment of data from a heterogeneous patient population together with further information regarding treatment practices among participating sites. In the US and EU, site level institutional review board or ethics committee approval must be in place prior to the initiation of recruitment to MEASuRE. All participating physicians must receive training on the study protocol prior to recruitment.

Because of the risk of anti-metreleptin antibodies with neutralising activity and risk of lymphoma during treatment, metreleptin is available in the US only through a restricted program under a risk evaluation and mitigation strategy (REMS) [14]. As such, metreleptin is dispensed in the US through a limited number of specialty pharmacies. Prescriber information (i.e., geographic location, specialty, and medical degree) and patient demographic characteristics are collected at these specialty pharmacies. Prescriber information provided by these specialty pharmacies is used to identify physicians who have prescribed metreleptin, after which they are invited by the sponsor to enroll in the registry if they have not already done so. Information from the specialty pharmacies is also used to identify the number of patients for whom each prescriber has written a metreleptin prescription.

In the EU, only prescribing physicians from countries where metreleptin is reimbursed and commercially available are invited to contribute data to MEASuRE. As all EU MEASuRE-specific data are collected via the ECLip registry, EU prescribing physicians must be an ECLip registry member prior to contributing data to MEASuRE.

### Patient enrolment and follow-up

At the time of registry enrolment, written consent is necessary for all participants per local requirements. This includes patients who can understand the requirements of the registry and provide written informed consent for themselves. For patients unable to respond on their own behalf, a parent or guardian provides consent. Pediatric patients may be included in all discussions, as appropriate.

To meet the requirements mandated by the FDA, MEASuRE endeavors to enroll at least 100 patients with GL treated with commercial metreleptin; patients enrolled from the US and EU will be used to achieve this target. All patients enrolled from the US will be followed for a minimum of ten years (i.e., from when the 100th enrolled patient reaches ten years of follow-up), unless they withdraw consent to participate in the registry, or die. EU patients will be enrolled and followed for the duration of the product lifecycle unless the patient withdraws consent or dies.

### Patient categories

All patients enrolled into the registry are categorized into one of two patient cohorts, i.e., a metreleptin new user cohort or a metreleptin prevalent user cohort. These cohorts are defined as follows:

- the *metreleptin new user cohort* includes patients who are initiating treatment with metreleptin, through commercial supply at the time of providing written consent for registry enrolment.
- the *metreleptin prevalent user cohort* includes patients who have been, or who are, treated with metreleptin through commercial supply but initiated treatment before registry enrolment and/or patients treated with metreleptin through commercial supply but after transferring their metreleptin treatment from a non-commercial source (e.g., clinical studies, expanded access programs).

The inclusion of patients who participated in clinical trials or an expanded access program will enable continued long-term assessment of patients beyond clinical trial participation. Furthermore, patients who received metreleptin, but later discontinued treatment will also be encouraged to enroll. It is recognized that patients who initiate metreleptin after it is commercially available but discontinue shortly thereafter may not be inclined to enroll in the registry since they are no longer receiving the product. These patients could differ in demographic characteristics from those who enroll and continue in the registry and may also have a different safety profile if they discontinued treatment due to an AE; thus, efforts will be made to enroll these patients.

For the prevalent metreleptin users, data collection will include data endpoints at baseline (e.g., demographics, medical history, and comorbidities at the time of initiation of metreleptin, medication history including concomitant medications and laboratory tests) and information on any serious AEs experienced during the six months prior to registry enrolment as part of baseline assessments. This will ensure that any primary endpoints that occur shortly after treatment initiation will not be underrepresented in the study population.

### Endpoints

MEASuRE collects data to determine the incidence and severity of AEs of special interest (AESIs) including pancreatic and hepatic AEs, hypoglycemia, hypersensitivity reactions, medication errors, serious and severe infections, loss of efficacy, autoimmune disorders, cancer, and deaths. MEASuRE also gathers data regarding demographic characteristics, key laboratory values and patterns of metreleptin use. Laboratory measurements (including glycated hemoglobin [HbA1c], fasting plasma glucose and serum triglycerides) will be assessed as effectiveness endpoints. Additional exploratory endpoints will assess the use of metreleptin in sub-populations and determine the effect of metreleptin on neuroendocrine, gonadal and thyroid hormones, brain development, bone metabolism, and sexual maturation. The incidence of anti-drug antibodies (ADAs) with blocking activity may be estimated using results from immunogenicity testing. A list of the endpoints assessed as part of the MEASuRE is provided in Table 1.

### Data collection and storage

US data are entered directly into MEASuRE via a web-based interface managed the clinical research organization (CRO, IQVIA) acting on behalf of the registry sponsor. At each specified bi-annual data collection time point, clinical information is abstracted from all patient visits including scheduled and/or unscheduled visits since the last data collection time point.

**Fig. 1:**
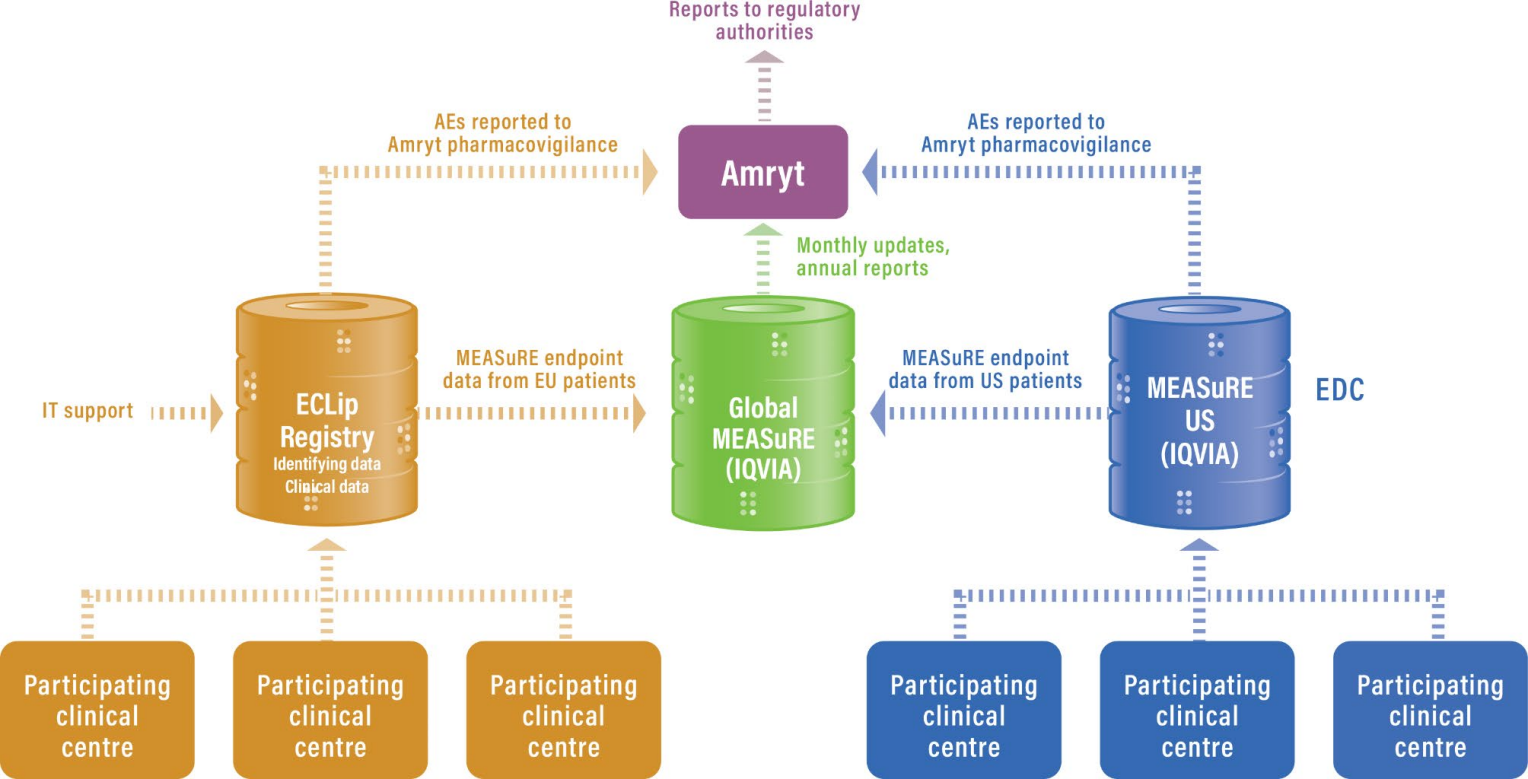
Arrangement of data flow into MEASuRE AE, adverse event; ECLip, European Consortium of Lipodystrophies; EDC, electronic data capture; MEASuRE, Metreleptin Efficacy And Safety Registry; EMA, European Medicines Agency; IT, information technology

In the EU, MEASuRE receives data from eligible patients via the ECLip registry, which collects data from participating sites through the ECLip registry portal (https://osse.epibio.uni-ulm.de/login.xhtml). Only MEASuRE-specific endpoint data collected via the ECLip registry from eligible patients who have given consent are extracted and transferred to MEASuRE; this process is supervised by ECLip registry coordinators based at Ulm University. Data are extracted on a tri-annual basis and forwarded to the CRO for integration into the global MEASuRE database, which aggregates US and EU-derived data (Fig. 1).

### Data confidentiality

To ensure patient confidentiality and to prevent data double-entry, patients are assigned a unique identifying number pseudonym for both the US and ECLip registry datasets. The key matching identification numbers with patient names are maintained by the participating sites, and only the unique identifier is recorded on the data collection forms with patient initials collected in the US (patient initials are not included in the data received via the ECLip registry). The patient identifiers are maintained if a patient is transferred to another study site post-enrolment. Upon enrolment, in localities where this is permissible, patients may be required, by their treating physician and/or site staff, to provide their name, phone and email contact information, and similar information for next of kin or guardians; however, this information is not collected in the global MEASuRE database. The treating physician and their site staff are required to securely store this information separately from other shared registry information. This information will only be used by the treating physician to obtain patient vital status and disposition if the patient becomes lost to follow-up.

In any presentations or publications of the results of the study, the patients’ identities will remain anonymous and confidential. The sponsor, its designee(s), and specific government health agencies may inspect the records of the study.

### Quality control

All sites are fully trained and are monitored through routine calls and visits, where necessary, with the CRO. Data obtained from patients are checked for accuracy prior to inclusion in MEASuRE reports submitted to regulatory authorities. In the US, this is primarily managed centrally by the CRO’s data management team in collaboration with the sites. For participating EU sites, the ECLip local operation group performs data reliability and completeness checks before forwarding data, tri-annually, to the CRO for inclusion in the MEASuRE database. The prescribing physicians participating in the registry are required to retain all study records and source documents for the maximum period required by applicable laws, regulations and guidelines, or institution procedures, or for the period specified by the sponsor, whichever is longer.

### Adverse event reporting

All safety information including non-serious AEs, serious AEs, AESIs (whether related to metreleptin or not), as well as pregnancies, AEs associated with maternal exposure, and pregnancy outcomes are documented in the electronic data capture system by the participating sites within 24 hours or one (1) business day of awareness to comply with local regulatory requirements.

### Interim reporting to regulatory authorities and future publications

All EU- and US-derived data submitted to MEASuRE are used to generate interim reports submitted to the FDA and the EMA, as mandated by these regulatory authorities. For peer-review publications involving data submitted to MEASuRE, the registry sponsor will be guided and advised by the steering committee as and when medically significant data are available and worthy of publication and dissemination to the wider medical community. In cases where a US or EU MEASuRE investigator(s), a MEASuRE steering committee member(s), or the MEASuRE registry sponsor wish to publish US-derived data from MEASuRE, requests must be made to and approved by the MEASuRE steering committee. For publications that include EU-derived data, the authors are also obliged to notify and receive approval from the ECLip registry board.

### Statistical analysis and sample size calculations

The safety analysis set for MEASuRE will contain all enrolled subjects who receive at least one commercial dose of metreleptin, regardless of the number of prior doses received. The effectiveness analysis set will contain all enrolled subjects who have at least one lab result beyond baseline lab results and received at least one commercial dose of metreleptin. Baseline data are defined as the latest available data prior to initiating metreleptin use (i.e., metreleptin naïve data).

For the US, the goal of the registry is to enroll at least 100 patients initiating treatment with metreleptin. The sample size calculation is based on patient recruitment experience in the clinical development program and published prevalence information, it is estimated that 20 patients per year will enroll in the registry. Assuming an annual loss to follow-up rate of 10%, the cumulative person-time information during the study period is 656 person-years. Given this expected number of person-years of exposure, the study would provide 95% assurance for the detection of at least one occurrence of a given, relatively rare major safety event with an incidence rate of approximately 0.46%, that is, an event that would occur approximately once in every 220 person-years of exposure. This calculation assumes independence of person-years. In addition, this sample size, on a per-person basis, would provide two-sided 95% confidence limits with a maximum width of approximately ± 8.8% for events that have a per-person incidence of 30% or less.

In the EU, patients are enrolled indefinitely for the duration of the lifecycle of the product, and as such, no power calculation was performed.

## Results

### The evolution of MEASuRE

**Fig. 2:**
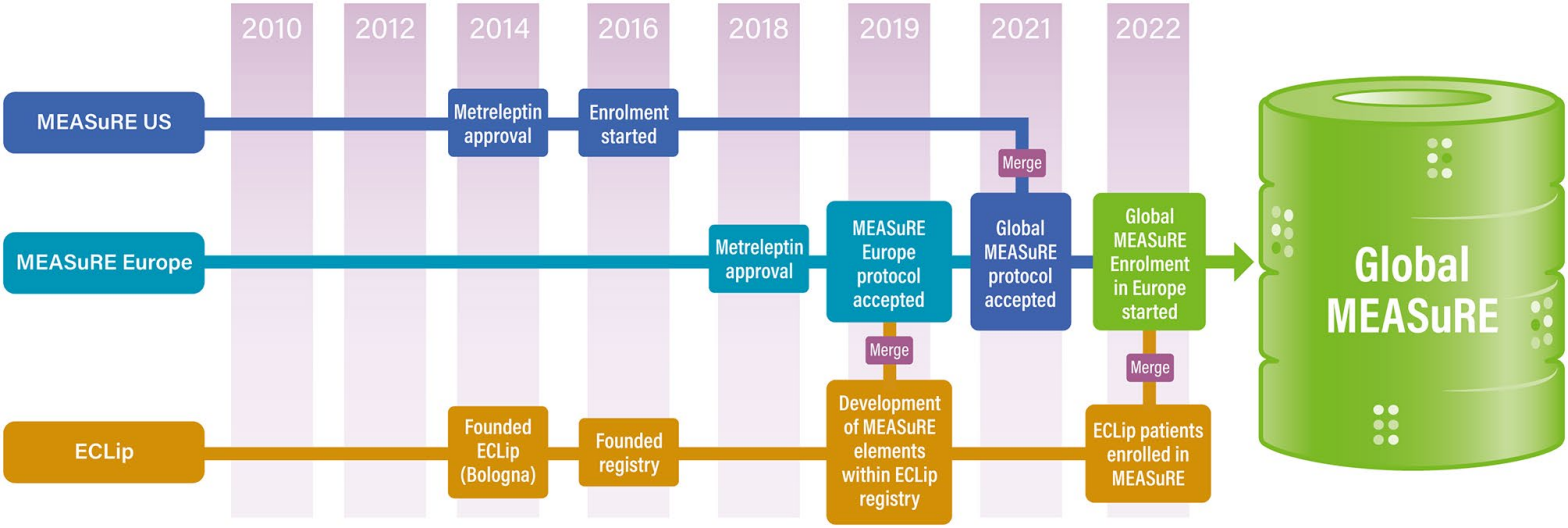
The history of the MEASuRE including the key protocol changes MEASuRE was initiated as a US FDA-mandated pharmacovigilance program. The EMA ECLip, European Consortium of Lipodystrophies; EMA, European Medicines Agency; FDA, Food & Drug Administration; MEASuRE, Metreleptin Efficacy And Safety Registry

Figure 2 details the evolution of MEASuRE including key protocol changes. The initial protocol for MEASuRE was prepared by the then market authorization holder, Amylin Pharmaceuticals (an Astra Zeneca company) in 2014 [34, 35]. Sponsorship of the registry moved to Aegerion Pharmaceuticals in January 2015 following its acquisition of metreleptin from Astra Zeneca and the first MEASuRE protocol was approved by the FDA that year. The EU MEASuRE protocol was prepared separately from the US protocol and approved by the EMA in 2019. Sponsorship of the registry in both regions transferred to Amryt Pharmaceuticals following its acquisition of Aegerion Pharmaceuticals in September 2019.

In February 2021, the protocols for the US and European registries were merged and harmonized to generate a single, global protocol, which was subsequently approved by the FDA and EMA. This step was undertaken to enable the aggregation of data from both regions and generate a dataset from a heterogenous global patient population that reflects the use of metreleptin in real-world settings.

In December 2021, an amendment was made to the global protocol following a request from the German health authorities that all eligible patients from Germany must be treated as per the EMA-approved product label; consequently, patients from Germany receiving metreleptin off-label are excluded from MEASuRE. The EU arm of MEASuRE was open to patient enrolment in January 2022.

### Collaboration with ECLip

A collaboration contract between Amryt Pharmaceuticals and the ECLip registry represented by Ulm University was negotiated and agreed upon, covering key points regarding the collection and transfer of data from the ECLip registry to MEASuRE. These included intellectual property, publication rights, data ownership, general data protection regulation (GDPR) processes, data quality checks, and final data transfer procedures. Contractual negotiations with the ECLip registry board were initiated by Aegerion Pharmaceuticals in late 2018 and were continued by Amryt Pharmaceuticals following the transfer of marketing authorization. A fully executed agreement was effective in August 2021 (approximately 2.5 years after negotiations began). While a core team at Amryt Pharmaceuticals and ECLip met frequently to discuss and progress the contract, each negotiation point was discussed and voted on by the ECLip registry board during their monthly meetings.

A key aspect of this collaboration was the broadening of the ECLip registry dataset to include additional effectiveness and safety endpoints specific to MEASuRE together with an AE reporting and reconciliation system. This procedure required a series of updates to the Open-Source Registry System for Rare Diseases [OSSE] [29, 36], a software platform funded by the German Federal Ministry of Health that supports the ECLip Registry. Thereafter, the system was tested and validated by both ECLip and Amryt Pharmaceuticals in compliance with the FDA’s Part 11 of Title 21 of the Code of Federal Regulations [CFR] [37]. Procedures, conducted by the local operating group of the ECLip registry, were established to assess the quality and reliability of MEASuRE-specific data extracted from the ECLip registry before forwarding these to the CRO. Additional information technology (IT) consulting resources were provided by the sponsor to support the ECLip registry such that data received by the CRO could be processed and combined with US-derived data. Finally, as the data elements collected from EU patients via the ECLip registry were recorded with different naming conventions versus US-derived data, a comprehensive data matching process was implemented, such that all data could be mapped, aggregated, and analyzed within MEASuRE regardless of the geographical source.

### Patient enrolment to date

Data were available on patient enrolment up 31st October 2022. Fifteen US sites (comprising 12 active sites and three sites no longer enrolling patients) and four EU sites (two in Germany and two in Italy; all active) have participated in the registry. The first US-based patient registered in October 2016, while the first patient from the EU registered in February 2022. Globally, 85 patients are enrolled in MEASuRE, 71 from the US and 14 from the EU. Of this global total, 67 patients have been treated for GL and 16 patients have been treated for PL; the lipodystrophy type of two additional patients enrolled from the US is currently unknown (Table 2).

**Table 2:** Lipodystrophy type and location of patients enrolled in MEASuRE as of 31st October 2022

	Patients with GL, n (%)	Patients with PL, n (%)
United States*	60 (89.6%)	9 (56.3%)
European Union	7 (10.4)	7 (43.7%)
Total	67	16

* The lipodystrophy type of two additional US patients is unknown

GL, generalized lipodystrophy; MEASuRE, Metreleptin Effectiveness and Safety Registry; PL, partial lipodystrophy

**Fig. 3:**
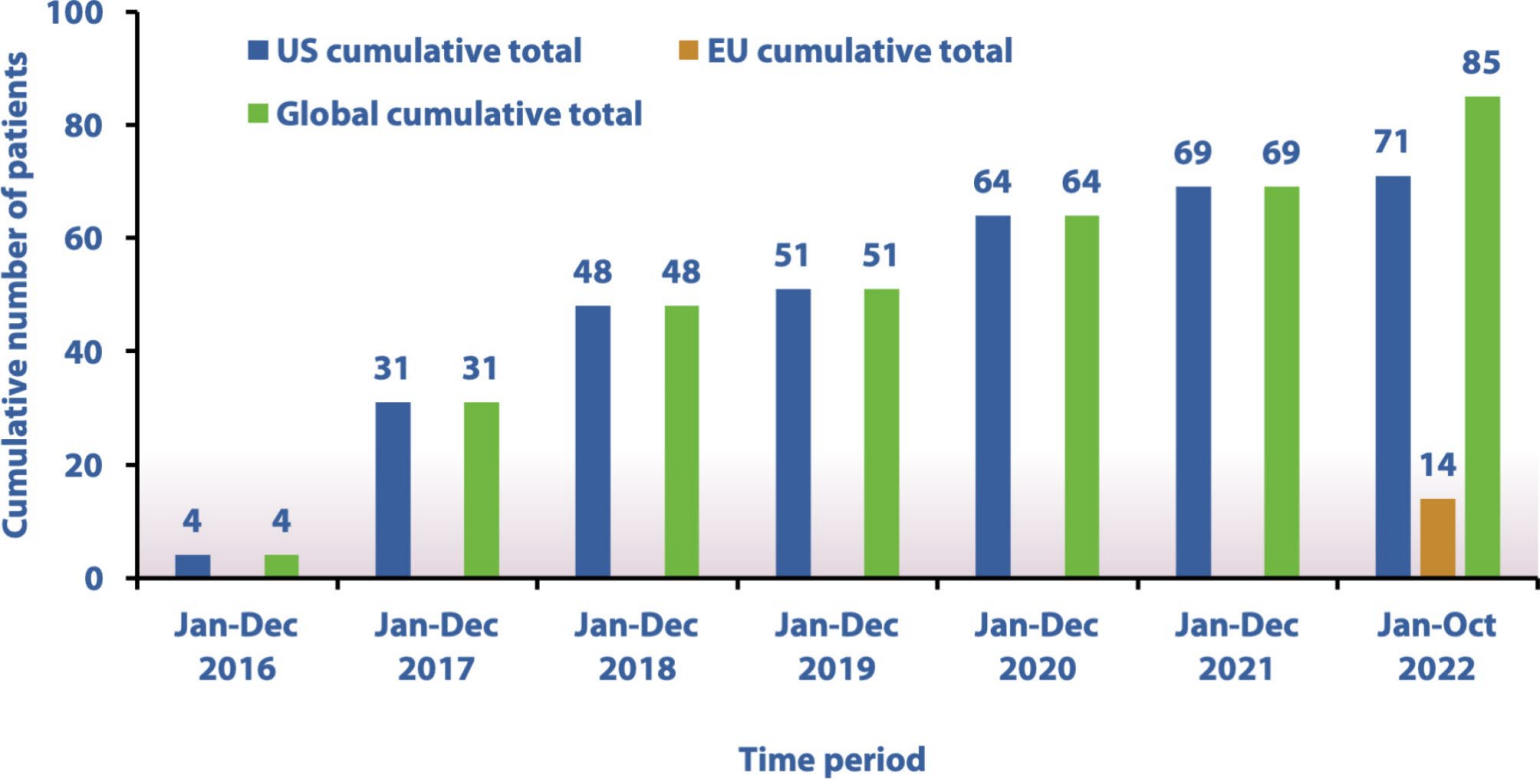
Patient accrual in MEASuRE

Cumulative patient enrolment is shown in Fig. 3. Four US patients were enrolled in 2016 with 27 patients enrolled in 2017, the highest yearly number thus far. US figures for each subsequent year are: 17 patients (2018), three patients (2019), 13 patients (2020), five patients (2021) and two patients (as of October 2022). All 14 EU patients were enrolled between February and October 2022, giving a global total for 2022 of 16 patients.

## Discussion

The low prevalence and broad genetic and phenotypic heterogeneity exhibited by patients presents challenges to elucidating the natural history and pathogenesis of lipodystrophy [2, 7]. These factors can limit the amount of available information to optimize treatment strategies for patients. Patient registries (which include disease and product registries) can help address these challenges by combining data from multiple sources to achieve sufficient sample sizes for clinical research and the assessment of diagnostic and therapeutic interventions [23, 24, 38].

Recent initiatives by the EMA and FDA have detailed the contribution of product registries to the study of rare diseases, and how they can support post-authorization safety studies (PASS) and post-authorization efficacy studies (PAES) [38–40]. These initiatives encourage collaboration between multiple stakeholders including registry sponsors, healthcare professionals, academic institutions, national regulatory agencies and, where applicable, pharmaceutical companies, to better understand key aspects of rare diseases including prevalence, natural history, associated complications, health burden and response to different management and treatment modalities. Where feasible, existing patient registries can reduce duplication of workload by leveraging established legal and regulatory frameworks, digital infrastructural resources, and methodological support [18, 25, 38].

MEASuRE was developed as a post-authorization requirement by the FDA and EMA to evaluate the long-term safety and effectiveness of metreleptin in routine clinical practice. Similar requirements have been requested of other medicinal products, including those indicated for use in rare disease. For example, Bouvy et al. (2017) showed that 31 registries (20 product registries, 11 disease registries) were requested for 30 of the 335 medicinal products (one product had a request for an adult and pediatric registry) receiving a positive opinion by the EMA’s Committee for Medicinal Products for Human Use (CHMP), between 2005 and 2013, as part of the marketing authorization process. Of these 31 registries, 29 (94%) had safety either as a primary or secondary objective, and 12 (39%) had effectiveness/efficacy as an objective. Furthermore, of the 30 medicinal products, 20 (67%) had an orphan indication [41]. These regulatory requests are consistent with the primary objectives of MEASuRE.

Low levels of involvement or inconsistent participation by eligible physicians and patients are key challenges for registries, often resulting in low patient accrual, missing or poor-quality data. As part of the analysis conducted by Bouvy et al. (2017), it was shown that of the 24 registries requested by the EMA which had initiated patient inclusion, 13 (54%) had a low patient accrual rate, while three (13%) had issues with poor data quality or missing data [41]. A follow-up analysis of these registries conducted by Pacurariu et al. (2018) revealed that 4 out of 14 ongoing registries had a patient accrual rate of less than 50% of their target [42]. Low patient accrual and inconsistent data can impact the value of the information collected by a product registry and can limit the statistical power and interpretation of the findings from subsequent analyses. It can also lead to a selection bias such that the patients enrolled in the registry do not fully represent the real-world population of treated patients [18]. The studies by Bouvy et al. (2017) and Pacurariu et al. (2018) also demonstrated that newly developed registries often experience a delayed time to start. For example, only 10 out of the 31 (32%) post-marketing surveillance registries requested by the EMA between 2005 and 2013 were finalized by 2017, with six of these having a median completion delay of 3 years [41, 42].

Several approaches can be adopted to enrich the data collected by product and disease registries and help address issues of low patient accrual and inconsistent data. For example, the development of global protocols can facilitate the collection of standardized data from different regions while meeting local regulatory requirements without the need for separate regional protocols [19, 43, 44]. In the case of MEASuRE, the approval of a global registry protocol harmonized the separate regulatory requirements of the FDA, EMA, and the German health authorities. It is anticipated that this global protocol will streamline the patient enrolment process and reduce the time needed the reach the target of least 100 patients with GL as per FDA requirements.

To further enhance the rate of patient enrolment, the decision was taken to collaborate with ECLip and use, where possible, their existing patient registry as supported by the EMA Patient Registries Initiative [18]. Through this collaboration, we have also leveraged the established processes and infrastructure of the ECLip registry, including clearly defined data collection methods, the provision of a meta-data repository, high-level data security measures, and data quality assessment procedures [22]. Important points agreed upon as part of our collaboration were data export parameters compliant with EU level and national GDPR, and data ownership, which remains with the participating sites and/or ECLip while enabling Amryt Pharmaceuticals to forward data to the regulatory authorities as necessary. In the interests of the medical community and the lipodystrophy patient population, we also agreed with ECLip the publication process should either party wish to publish data related to or including MEASuRE-specific endpoints. As such, MEASuRE-specific data collected via the ECLip registry may be made available for other areas of lipodystrophy research.

Following lengthy negotiations with the ECLip registry board, MEASuRE is now fully operational and has the capability of collecting data from a wide geographical network of participating European sites via the ECLip registry’s single central location. The benefits of this strategy include a reduction in the time and costs associated with registry set-up and maintenance [20, 45], improved site participation due to familiarity with the ECLip registry interface, and the removal of the need to implement data collection resources and procedures with individual sites.

It should be noted that the integration of MEASuRE-specific endpoints into the ECLip registry presented several technical challenges for both registries. First, modification of the ECLip registry IT framework was needed to add new information specific to MEASuRE such as additional safety and effectiveness endpoints, an independent AE reporting system and a supporting AE reconciliation process. As this was ECLip’s first non-academic collaboration, updates of this nature were novel and required the development of computational and systematic processes, including testing the functionality of the OSSE software, to ensure the data captured achieved the desired outcomes for MEASuRE. After several rounds of updates, the OSSE software was assessed for compliance with the FDA’s 21 CFR Part 11. Second, it has been reported that heterogeneity in the data collected by different registries can hinder the development of a single, common repository [20, 46]. A standardized approach that uses a common data entry format and naming system to facilitate data aggregation from multiple registries should be implemented, where feasible; however, this is unlikely to be achievable in many settings. In the case of MEASuRE, data elements collected via the ECLip registry were recorded with different naming conventions compared with US-derived data. Consequently, a comprehensive data matching exercise was conducted such that data are reliably pooled and assessed within MEASuRE regardless of the geographical source. While these processes involved a considerable investment of time and costs, they resulted in the development of a robust data collection system with the capacity for gathering and integrating standardized US- and EU-derived data.

The first EU patient was enrolled into MEASuRE in February 2022 and since then a total of 14 EU patients have registered giving a cumulative global total of 16 patients over a ten-month period up to October 2022. This is comparable to the total number enrolled for 2018 (17 US patients), the second highest ranked year for enrolment to the registry. This finding lends some support in using existing registries and broadening the geographical scope of MEASuRE to expedite patient enrolment timelines and improve the efficiency of data collection. This approach also ensures that the registry better represents the global metreleptin-treated population.

Notably, there was a substantial lag time between the date of metreleptin marketing authorization in the US and EU and the inclusion of the first patients from these regions to MEASuRE (over 2.5 years and over 3.5 years, respectively). A lag time between product authorization and first patient enrolment has been reported for other product registries [18, 41, 42]. Possible contributory factors to this lag time for MEASuRE include the review and approval cycles with the FDA and EMA to achieve a global protocol; the submission and approval of the study at the national and site level; the negotiation of contracts at each participating clinical site in both the US and EU; the gathering of patient-informed consent; the willingness of patients and sites to participate in the registry; and delayed product launches in EU countries after central marketing authorization has been granted. Other factors contributing to MEASuRE’s delayed onset of enrolment include the negotiating period with ECLip and the transition of metreleptin marketing authorization, and therefore responsibility of the registry, between three pharmaceutical companies between late 2014 and late 2019. Reduced patient contact with specialist centers during the coronavirus disease 2019 (COVID-19) pandemic may have also impacted enrolment. Indeed, the COVID-19 pandemic highlights the need for registry protocols that can adapt to unforeseen circumstances in which patient contact and/or travel may be restricted. Despite these challenges, MEASuRE has successfully enrolled 85 patients to date, reflecting the ongoing commitment of physicians and patients to improve the understanding of the safety and effectiveness of metreleptin in lipodystrophy.

The development of MEASuRE demonstrates that collaborative approaches between the coordinators of disease and post-authorization product registries is feasible and may be a necessary step for improving the knowledge of rare diseases. Based on our experience, we recommend that any current and future endeavors to develop post-authorization product registries for rare diseases seek, where possible, to use existing registries. The linking of a new registry with an existing registry should occur as early as possible in the product life cycle to maximize data collection opportunities. We fully acknowledge that the integration of new and old registries can be a lengthy process requiring the provision and sharing of resources and a high level of commitment and engagement from both sides; for example, for MEASuRE, weekly calls were scheduled for over one year between ECLip board members and representatives of Amryt Pharmaceuticals. Therefore, the sponsors of new registries adopting this approach need to build in sufficient time not only to negotiate but also implement and validate any infrastructural changes to the existing registry. It is conceivable that other projects involving the collaboration between a new registry with multiple existing registries could further extend these timelines. Regulatory authorities should be notified of the intention to collaborate as soon as possible to facilitate review and approval processes. As illustrated here, collaborating with existing registries offers an opportunity to reduce registry set-up time and costs, enhance patient enrolment, and expedite data collection and sharing processes for mutual benefit. These approaches can help nurture important relationships between a broad range of stakeholders (e.g., researchers, physicians, patient associations, medicine regulators and pharmaceutical companies) to deliver sustainable registries that improve the knowledgebase of rare diseases and their treatment.

Looking forward, the next steps for MEASuRE include the enrolment of patients from additional countries, within the EU and worldwide, as sites register with ECLip and as reimbursement of metreleptin is achieved. For example, in England and Wales, metreleptin was approved for reimbursement in 2021 by the National Institute for Health and Care Excellence (NICE) for use as an adjunct to diet for the treatment of the metabolic complications of GL and PL [47]. As with data from the EU, United Kingdon (UK)-derived data will be collected via the ECLip registry; this is contingent on regulatory and ethical approval of ECLip, and subsequent approval of MEASuRE, to operate in the UK. UK sites can then be opened for enrolment—this is anticipated to occur in early 2023. Also, MEASuRE will be established in countries outside of the EU and US in accordance with local regulations and obligations following commercial reimbursement (e.g., Brazil). Countries outside the EU will follow the US model for data collection.

## Conclusion

Here, we have outlined the key challenges encountered and addressed during the development of a new product post-authorization registry, MEASuRE, which evaluates the safety and effectiveness outcomes for the metreleptin-treated population. Primary among these was the linking of MEASuRE with the existing ECLip registry; this required the expansion of the ECLip registry framework to collect MEASuRE-specific endpoints, extensive data matching processes to ensure data consistency from EU and US sources, and rigorous data validation following the pooling of global data. This integration process was lengthy and delayed the collection of EU-derived data. Other factors that may to have contributed to the delay in EU patient enrolment include the transition in MEASuRE’s sponsorship across three pharmaceutical companies since 2014 and the COVID-19 pandemic resulting in reduced patient engagement. Despite these challenges, we posit that feasibility of EU patient enrolment will be improved through the supports provided through our collaboration with ECLip.

We believe that the learnings taken from the establishment of MEASuRE may have utility beyond lipodystrophy. Following the publication of the EMA’s Patient Registry Initiative, many orphan medications may require post-approval commitments involving disease and product registries to achieve marketing authorization. In this regard, we envisage that the challenges and solutions detailed here will assist those embarking on similar enterprises and reduce duplications in effort and funding.

## Data Availability

No data are available from MEASuRE at this time.

## List of abbreviations

AE: adverse event
ADA: anti-drug antibody
AESI: adverse event of special interest
AGL: acquired generalized lipodystrophy
APL: acquired partial lipodystrophy
CGL: congenital lipodystrophy
CHMP: Committee for Medicinal Products for Human Use
COVID-19: COVID-19
CRO: clinical research organization
ECLip: European Consortium of Lipodystrophies
EMA: European Medicines Agency
EMR: electronic medical records
EU: European Union
EuRRECa: European Registries for Rare Endocrine Conditions
FAIR: findable, accessible, interoperable, reusable
FDA: Food and Drug Administration
FPLD: familial partial lipodystrophy
GDPR: General Data Protection Regulation
GL: generalized lipodystrophy
HbA1c: glycated hemoglobin
HIV: human immunodeficiency virus
LD: lipodystrophy
MEASuRE: Metreleptin Effectiveness And Safety Registry
NAFLD: Non-alcoholic fatty liver disease
NIH: National Institutes of Health
OSSE: Open Source Registry System for Rare Diseases
PAES: Post-authorization efficacy studies
PASS: Post-authorization safety studies
PL: partial lipodystrophy
REMS: Risk evaluation and mitigation strategy
SmPC: summary of product characteristics
IT: Information technology
UK: United Kingdom
US: United States of America

## References

[1] Brown RJ, Araujo-Vilar D, Cheung PT, Dunger D, Garg A, Jack M (2016). The diagnosis and management of Lipodystrophy Syndromes: a Multi-Society Practice Guideline. J Clin Endocrinol Metab.

[2] Garg A (2004). Acquired and inherited lipodystrophies. N Engl J Med.

[3] Araujo-Vilar D, Santini F (2019). Diagnosis and treatment of lipodystrophy: a step-by-step approach. J Endocrinol Invest.

[4] Lightbourne M, Brown RJ (2017). Genetics of Lipodystrophy. Endocrinol Metab Clin North Am.

[5] Akinci B, Meral R, Oral EA (2018). Phenotypic and genetic characteristics of Lipodystrophy: pathophysiology, metabolic abnormalities, and comorbidities. Curr Diab Rep.

[6] Garg A (2011). Clinical review: lipodystrophies: genetic and acquired body fat disorders. J Clin Endocrinol Metab.

[7] Chiquette E, Oral EA, Garg A, Araujo-Vilar D, Dhankhar P (2017). Estimating the prevalence of generalized and partial lipodystrophy: findings and challenges. Diabetes Metab Syndr Obes.

[8] Gonzaga-Jauregui C, Ge W, Staples J, Van Hout C, Yadav A, Colonie R (2020). Clinical and molecular prevalence of Lipodystrophy in an unascertained large clinical care cohort. Diabetes.

[9] Akinci B, Oral EA, Neidert A, Rus D, Cheng WY, Thompson-Leduc P (2019). Comorbidities and survival in patients with lipodystrophy: an International Chart Review study. J Clin Endocrinol Metab.

[10] Adams C, Stears A, Savage D, Deaton C (2018). We’re stuck with what we’ve got”: the impact of lipodystrophy on body image. J Clin Nurs.

[11] Martin SA, Sanchez RJ, Olayinka-Amao O, Harris C, Fehnel S (2022). Qualitative interviews in patients with lipodystrophy to assess the patient experience: evaluation of hunger and other symptoms. J Patient Rep Outcomes.

[12] Fourman LT, Grinspoon SK (2022). Approach to the patient with Lipodystrophy. J Clin Endocrinol Metab.

[13] Zammouri J, Vatier C, Capel E, Auclair M, Storey-London C, Bismuth E, et al. Molecular and Cellular Bases of Lipodystrophy Syndromes. Front Endocrinol (Lausanne). 2021;12. 10.3389/fendo.2021.803189. 10.3389/fendo.2021.803189 PMC8763341 35046902

[14] Amryt Pharmaceuticals DAC. Myalept package insert 2022. Available from: https://www.accessdata.fda.gov/drugsatfda_docs/label/2022/125390s024lbl.pdf [accessed April [accessed April 2023].023]

[15] Amryt Pharmaceuticals DAC. Myalepta summary of product characteristics 2021. Available from: https://www.ema.europa.eu/en/medicines/human/EPAR/myalepta [accessed April [accessed April 2023].023]

[16] Brown RJ, Oral EA, Cochran E, Araujo-Vilar D, Savage DB, Long A (2018). Long-term effectiveness and safety of metreleptin in the treatment of patients with generalized lipodystrophy. Endocrine.

[17] Oral EA, Gorden P, Cochran E, Araujo-Vilar D, Savage DB, Long A (2019). Long-term effectiveness and safety of metreleptin in the treatment of patients with partial lipodystrophy. Endocrine.

[18] European Medicines Agency. Guideline on registry-based studies Amsterdam 2021 [updated 19 October 2022; cited 2022 19 October]. Available from: https://www.ema.europa.eu/en/guideline-registry-based-studies-0 [accessed April [accessed April 2023].023]

[19] Forrest CB, Bartek RJ, Rubinstein Y, Groft SC (2011). The case for a global rare-diseases registry. Lancet.

[20] Olmo CA, McGettigan P, Kurz X (2019). Barriers and Opportunities for Use of Patient Registries in Medicines Regulation. Clin Pharmacol Ther.

[21] Boulanger V, Schlemmer M, Rossov S, Seebald A, Gavin P (2020). Establishing patient registries for Rare Diseases: Rationale and Challenges. Pharmaceut Med.

[22] Gliklich RE, Dreyer NA, Leavy MB. Registries for Evaluating Patient Outcomes: A User’s Guide [Internet]. 3rd edition 2014. Available from: https://www.ncbi.nlm.nih.gov/books/NBK208643/ [accessed April [accessed April 2023].023]

[23] Kolker S, Gleich F, Mutze U, Opladen T (2022). Rare disease registries are key to evidence-based Personalized Medicine: highlighting the european experience. Front Endocrinol (Lausanne).

[24] Jonker CJ, Bakker E, Kurz X, Plueschke K (2022). Contribution of patient registries to regulatory decision making on rare diseases medicinal products in Europe. Front Pharmacol.

[25] Food and Drug Administration. Real-World Data: Assessing Registries to Support Regulatory Decision-Making for Drug and Biological Products Guidance for Industry 2021 [Available from: http://www.fda.gov/Drugs/GuidanceComplianceRegulatoryInformation/Guidances/ucm259809.htm [accessed April [accessed April 2023].023]

[26] Food and Drug Administration. Framework for FDA’s Real-World Evidence Program 2018 [Available from: https://www.fda.gov/media/120060/download, accessed April 2023].

[27] European Medicines Agency. Patient Registry Initiative- Strategy and Mandate of the Cross-Committee Task Force London 2017 [Available from: https://www.ema.europa.eu/en/documents/other/patient-registry-initiative-strategy-mandate-cross-committee-task-force_en.pdf, accessed April 2023].

[28] European Consortium of Lipodystrophies (ECLip). Lipodystrophies 2019. Available from: https://www.eclip-web.org/lipodystrophies/ [accessed April [accessed April 2023].023]

[29] Schaaf J, Kadioglu D, Goebel J, Behrendt CA, Roos M, van Enckevort D (2018). OSSE goes FAIR - implementation of the FAIR Data Principles for an Open-Source Registry for Rare Diseases. Stud Health Technol Inform.

[30] von Schnurbein J, Adams C, Akinci B, Ceccarini G, D’Apice MR, Gambineri A (2020). European lipodystrophy registry: background and structure. Orphanet J Rare Dis.

[31] Public Policy Committee ISoP (2016). Guidelines for good pharmacoepidemiology practice (GPP). Pharmacoepidemiol Drug Saf.

[32] European Medicines Agency. ICH E6 (R2) Good clinical practice - Scientific guideline2002. Available from: https://www.ema.europa.eu/en/ich-e6-r2-good-clinical-practice-scientific-guideline [accessed April [accessed April 2023].023]

[33] World Medical Association (2013). World Medical Association Declaration of Helsinki: ethical principles for medical research involving human subjects. JAMA.

[34] ClinicalTrials.gov. History of Changes for Study: NCT02325674: MEASuRE: Metreleptin Effectiveness And Safety Registry (MEASuRE)2022. Available from: https://clinicaltrials.gov/ct2/history/NCT02325674 [accessed April [accessed April 2023].023]

[35] AstraZeneca, US FDA approves orphan drug MYALEPT™ (metreleptin for injection). 2014. Available from: https://www.astrazeneca.com/media-centre/press-releases/2014/us-fda-approved-myalept-lepti-deficiency-treatment-25022014.html#, accessed April 2023.

[36] OSSE – Open Source Registry System for Rare. Diseases [cited 2022 15 November]. Available from: https://en.osse-register.de/en/; accesed January 2023.

[37] Food and Drug Administration. Guidance for Industry: Part 11, Electronic Records; Electronic Signatures - Scope and Application 2003 [Available from: https://www.fda.gov/regulatory-information/search-fda-guidance-documents/part-11-electronic-records-electronic-signatures-scope-and-application; accessed April 2023.

[38] McGettigan P, Alonso Olmo C, Plueschke K, Castillon M, Nogueras Zondag D, Bahri P (2019). Patient registries: an underused resource for Medicines evaluation: operational proposals for increasing the use of patient registries in regulatory assessments. Drug Saf : Int J Med Toxicol drug experience.

[39] European Medicines Agency. Scientific guidance on post-authorisation efficacy studies London 2015 [Available from: https://www.ema.europa.eu/en/documents/scientific-guideline/scientific-guidance-post-authorisation-efficacy-studies-first-version_en.pdf; accessed April 2023].

[40] European Medicines Agency. Guideline on good pharmacovigilance practices (GVP) Module VIII – Post-authorisation safety studies (Rev 3) 2017 [Available from: https://www.ema.europa.eu/en/documents/scientific-guideline/guideline-good-pharmacovigilance-practices-gvp-module-viii-post-authorisation-safety-studies-rev-3_en.pdf; accessed April 2023].

[41] Bouvy JC, Blake K, Slattery J, De Bruin ML, Arlett P, Kurz X (2017). Registries in european post-marketing surveillance: a retrospective analysis of centrally approved products, 2005–2013. Pharmacoepidemiol Drug Saf.

[42] Pacurariu A, Plueschke K, Olmo CA, Kurz X (2018). Imposed registries within the european postmarketing surveillance system: extended analysis and lessons learned for regulators. Pharmacoepidemiol Drug Saf.

[43] European Medicines Agency. Initiative for patient registries – strategy and pilot phase2015. Available from: https://www.ema.europa.eu/en/documents/other/initiative-patient-registries-strategy-pilot-phase_en.pdf [accessed April [accessed April 2023].023]

[44] Nicholson N, Perego A (2020). Interoperability of population-based patient registries. J Biomed Inform.

[45] European Medicines Agency. Patient Registries [Available from: https://www.ema.europa.eu/en/human-regulatory/post-authorisation/patient-registries [accessed April [accessed April 2023].023]

[46] Mordenti M, Boarini M, D’Alessandro F, Pedrini E, Locatelli M, Sangiorgi L (2022). Remodeling an existing rare disease registry to be used in regulatory context: Lessons learned and recommendations. Front Pharmacol.

[47] Amryt Pharmaceuticals DAC. Myalepta summary of product characteristics 2022. Available from: https://www.medicines.org.uk/emc/product/11184/smpc [accessed April [accessed April 2023].023]

